# WO_3_/W Nanopores Sensor for Chemical Oxygen Demand (COD) Determination under Visible Light

**DOI:** 10.3390/s140610680

**Published:** 2014-06-17

**Authors:** Xuejin Li, Jing Bai, Qiang Liu, Jianyong Li, Baoxue Zhou

**Affiliations:** School of Environmental Science and Engineering, Shanghai Jiao Tong University, No. 800 Dongchuan Rd, Shanghai 200240, China; E-Mails: lxj2006333015@163.com (X.L.); bai_jing@sjtu.edu.cn (J.B.); linsanityq@163.com (Q.L.); joe9731@126.com (J.L.)

**Keywords:** WO_3_ sensor, visible light, COD

## Abstract

A sensor of a WO_3_ nanopores electrode combined with a thin layer reactor was proposed to develop a Chemical Oxygen Demand (COD) determination method and solve the problem that the COD values are inaccurately determined by the standard method. The visible spectrum, e.g., 420 nm, could be used as light source in the sensor we developed, which represents a breakthrough by limiting of UV light source in the photoelectrocatalysis process. The operation conditions were optimized in this work, and the results showed that taking NaNO_3_ solution at the concentration of 2.5 mol·L^−1^ as electrolyte under the light intensity of 214 μW·cm^−2^ and applied bias of 2.5 V, the proposed method is accurate and well reproducible, even in a wide range of pH values. Furthermore, the COD values obtained by the WO_3_ sensor were fitted well with the theoretical COD value in the range of 3–60 mg·L^−1^ with a limit value of 1 mg·L^−1^, which reveals that the proposed sensor may be a practical device for monitoring and controlling surface water quality as well as slightly polluted water.

## 1. Introduction

Chemical oxygen demand (COD) is considered to be a quite significant parameter in contemporary society. The traditional COD measurement, the potassium dichromate oxidation method, has been applied for decades but is unsuitable to determine samples with COD concentrations lower than 50 mg·L^−1^. The potassium permanganate oxidation (COD_Mn_) method, another standard method for determining low COD concentrations within the range of 5–50 mg·L^−1^, however, does not accurately reflect the COD values of samples for its poor oxidation ability. Furthermore, both methods suffer shortcomings such as being time-consuming, high cost, and using toxic chemicals in the determination process [1]. Considering this situation, it is urgent to propose a method which could accurately determine the COD values of aquatic samples.

Sensors have been used to solve various issues [2–4], and in this kind of case a semiconductor electrode sensor combined with a thin layer reactor could serve as a COD determination device which requires only 1–5 min to complete an examination and consumes very limited amounts of reagent (electrolyte only) when applying the photoelectrocatalysis approach to generate electron/hole pairs to degrade organic matter on the semiconductor surface. Among semiconductors [5,6], TiO_2_ nanomaterials have attracted special attention [7–9] due to its superior oxidative abilities under UV illumination and is typically non-toxic, inexpensive and environmentally benign. However, two drawbacks have limited the application of TiO_2_ nanomaterials: one is the limiting use of the light source for the wide band gap of 3.2 eV [9] which could only use the UV spectrum as a light source to excite the photogenerated electron/hole pairs; the other one is the unexpected heating of the short wave light source during the degradation process.

In this work, a new kind of sensor, a WO_3_ nanopores electrode, in conjunction with a thin layer reactor was proposed for application in COD determination. Benefitting from the narrow band gap (2.6 eV) of WO_3_ [10–12], a light source in the visible light spectrum could excite the photogenerated electron/hole pairs. In addition, like TiO_2_, WO_3_ is low cost and non-toxic. The WO_3_ electrode was prepared via a facile electrochemical anodization method from tungsten foil with a nanoporous structure which assured its good stability. Compared to previous WO_3_ electrodes, the as-prepared WO_3_ nanoporous electrode displays excellent mechanical stability and possesses larger specific surface area which enhances its light harvesting capability and electrolyte contacting. Due to all of the above-mentioned factors, the WO_3_ sensor represents an improvement in the phototelectrocatalytic degradation of organics.

## 2. Experimental Section

All the reagents, purchased from Sinopharm Chemical Reagent Co., Ltd., (Shanghai, China) were analytical reagent grade and used without further purification unless otherwise stated. Deionized water was used for the solution preparation. NaNO_3_ solution served as supporting electrolyte for the COD determination in the photoelectrocatalysis method.

The WO_3_/W nanopores electrode sensor was prepared by an electrochemical anodic oxidation method in which tungsten was served as anode and platinum as cathode. In a typical process, the preparation was conducted in a mixture solution of 0.2 wt % NaF and 0.3% HF at 60 V for 1 h and subsequently at 40 V for 30 min. The resulting sample was annealed in an air atmosphere at 450 °C for 3 h. The surface morphology of the WO_3_ film was investigated by field emission scanning electron microscopy (FE-SEM FEI-Sirion200, Hillsboro, America) under a voltage of 5 kV.

**Figure 1.**
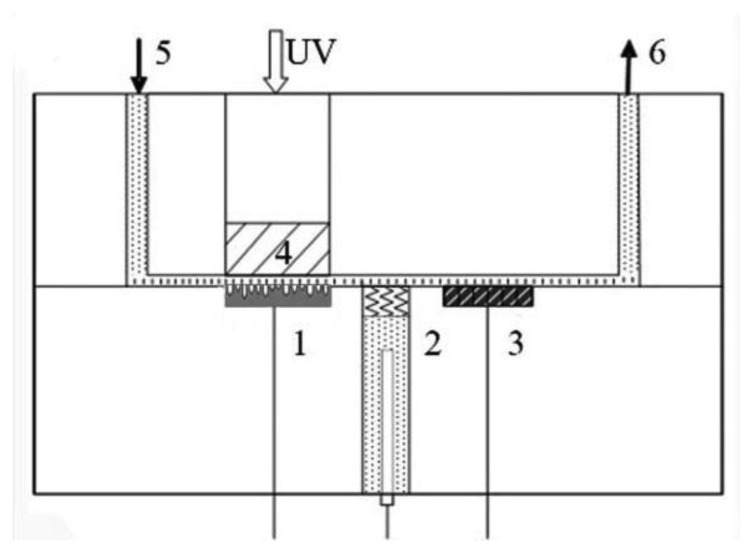
Schematic depiction of the thin layer reactor based on a WO_3_ sensor. (1) WO_3_/W nanopores sensor; (2) saturated Ag/AgCl reference electrode; (3) Pt counter electrode; (4) quartz window; (5) flow inlet; (6) flow outlet.

The photoelectrochemical experiment was conducted in a thin layer reactor as shown in Figure 1, in which the thickness was 0.1 mm and diameter was 1 cm. A typical three-electrode system was applied with a platinum foil counter electrode, a saturated Ag/AgCl reference electrode and a WO_3_ nanopores working electrode under visible light illumination at a wavelength of 420 nm. The solution was injected into the reactor by an automatic injector purchased from Xi'an Remex Analysis Instrument Company (Xi'an, China). The potential and current of the working electrode were controlled by an electrochemical workstation (CHI 610D, Chenghua, Shanghai, China).

## 3. Results and Discussion

The phototelectrocatalysis oxidation process of organic compounds in the WO_3_ nanopores electrode sensor can be represented by the following formula:

(1) $${\text{C}}_{\text{y}}{\text{H}}_{\text{m}}\;{\text{O}}_{\text{j}}{\text{N}}_{\text{k}}{\text{X}}_{\text{q}}+\left(2\text{y}-\text{j}\right){\text{H}}_{2}\text{O}\to {\text{yCO}}_{2}+{\text{qX}}^{-}+{\text{kNH}}_{3}+\left(4\text{y}-2\text{j}+\text{m}-3\text{k}\right){\text{H}}^{+}+\left(4\text{y}-2\text{j}+\text{m}-3\text{k}-\text{q}\right){\text{e}}^{-}$$

where N and X represents a nitrogen and a halogen atom, respectively, and y, m, j, k and q represent the numbers of carbon, hydrogen, oxygen, nitrogen and halogen atoms, respectively.

**Figure 2.**
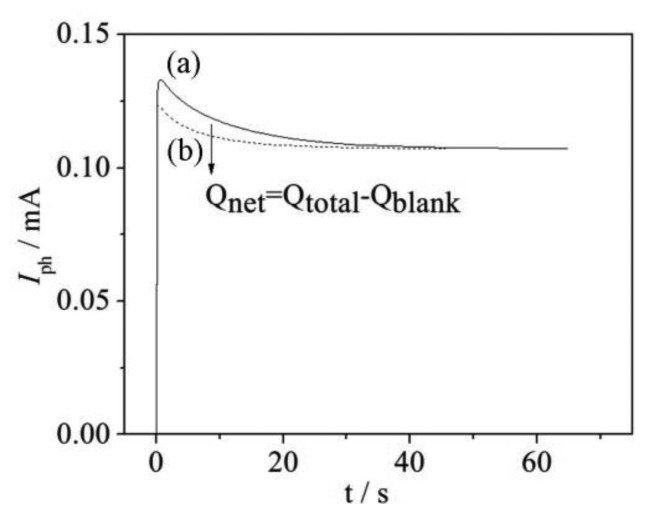
Typical photocurrent responses of sample containing organics: (**a**) and none organics; (**b**) under illumination of visible light at 420 nm.

Faraday's law can be used to quantify the concentration by measuring the charge generated by organic compounds degradation:

(2) $$Q_{\text{net}}=\int I_{\mathrm{ph}}=\text{nFVC}$$

where *I*_ph_ represents the organic compounds degradation photocurrent, *n* represents the amount of transferred electron generated by unit mole organic oxidation, *F* is the Faraday constant, *V* and *C* is the sample volume and concentration, respectively. From Equation (2) it can be inferred that the experimental *Q*_net_ could be calculated from the photocurrent degradation curves obtained by the WO_3_ sensor (see Figure 2), in other words, the net charge generated by the degradation of organic compounds could be calculated by subtracting the background value (*Q*_blank_) from the total net charge (*Q*_total_).

Besides, according to the definition of COD and the chemical reaction formula:

(3) $${\text{O}}_{2}+4{\text{H}}^{+}+4{\text{e}}^{-}\to 2{\text{H}}_{2}\text{O}$$

The quantity of transferred electrons could be converted to that of oxygen consumption and does not depend on the type of organic matter, which has been proved in our previously work [9].

(4) $$\text{COD}=\frac{nc}{4}\times 32000=\frac{Q_{\text{net}}}{4FV}\times 32000=KQ_{\text{net}}$$

### 3.1. Characterization of WO_3_ Nanopores Electrode Sensor

**Figure 3.**
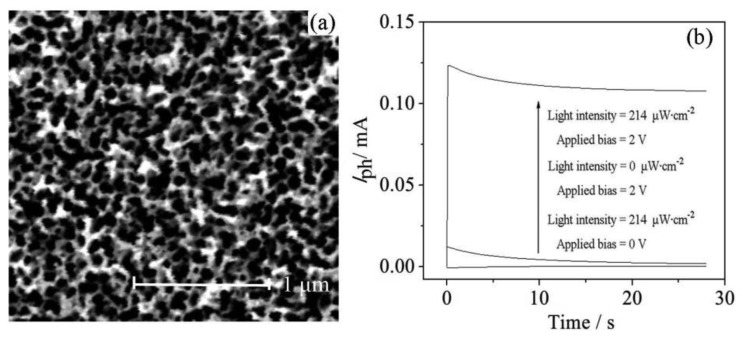
(**a**) SEM image of WO_3_/W nanopores; (**b**) The response of the WO_3_ sensor under different conditions.

In order to improve the separation of photoelectron/hole pairs, the WO_3_/W electrode properties should be optimized. It can be seen from the scanning electron microscopy (SEM) image (Figure 3a) that continuous nanopores with a diameter of 55∼100 nm covered the tungsten surface and the nanopores directly grew on the tungsten, which made the electrons easily exported to the external circuit.

Nanopores with a large surface area contacting the electrolyte produce more reaction sites to promote the reaction efficiency. Figure 3b shows the *I*–*t* curves obtained by the WO_3_/W nanopores sensor under photoelectrocatalysis, electrocatalysis and photocatalysis conditions, respectively. It can be seen that the WO_3_/W nanopores electrode performed well when both light illumination and bias were applied, and the photocurrent values are obviously higher than with the other two methods (electrocatalysis and phototcatalysis) under these photoelectrocatalysis conditions. In addition, it can be seen that the resulting electrode possesses good visible light response, good mechanical stability and large surface area, which would be beneficial to the separation of photoelectron/hole pairs.

### 3.2. Effect of the Supporting Electrolyte

**Figure 4.**
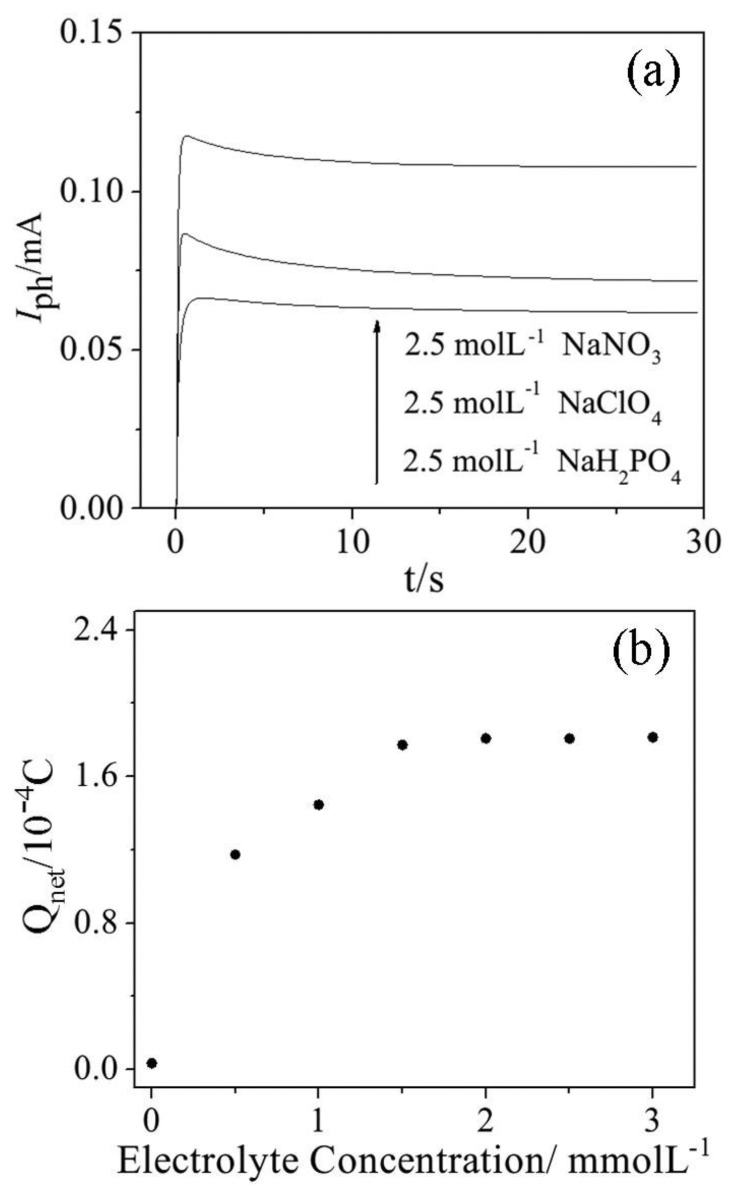
(**a**) Photocurrent responses of different electrolyte solutions without organic; (**b**) Effect of electrolyte concentrations on Q_net_ determination.

The supporting electrolyte is crucial to transfer electrons from the sensor to the external circuit because the quantity of the captured charge directly affects the COD determination results according the determination principle. In order to choose a suitable electrolyte and concentration, three kinds of sodium salts were tested in the photoelectrocatalysis process. As shown in Figure 4a, both the maximum value of the photocurrent response signals and the shortest time (about 20 s) to achieve the steady state were realized by using 2.5 mol·L^−1^ NaNO_3_ as the supporting electrolyte.

The various concentrations of NaNO_3_ electrolyte were tested taking glucose with a known theoretical COD (ThCOD) value of 42 mg·L^−1^ as target sample. The results shown in Figure 4b illustrate that when the electrolyte concentrations are in the range of 2 to 3 mol·L^−1^, the determined Q_net_ values show good stability and are obviously higher than those obtained under the condition that the electrolyte concentrations are lower than a certain value, e.g., 1.5 mol·L^−1^. In order to make sure the electrons pass the narrow pathway of the sensor to external circuit, 2.5 mol·L^−1^ NaNO_3_ was chosen as the supporting electrolyte for the subsequent experiments.

### 3.3. Effect of the Light Intensity

**Figure 5.**
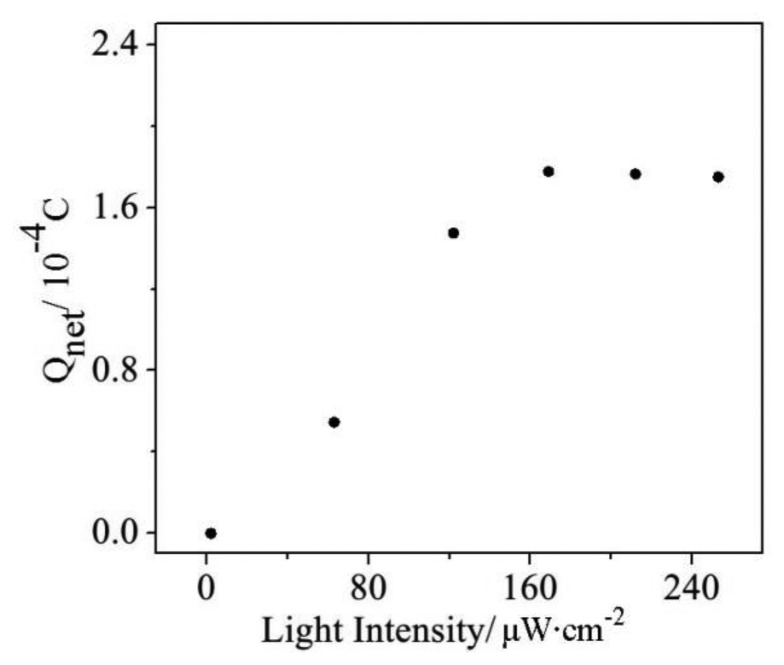
Effect of the light intensity on Q_net_ determination.

The light source is very important in the photoelectrocatalysis process because the light excites the photoelectron/hole pairs on the surface of WO_3_/W electrode. The light-excited photoelectron holes can then oxidize the organics. As shown in Figure 5, the obtained Q_net_ values are much smaller when the light intensity is weaker than 170 μW·cm^−2^ because the weak light intensity excites only a small amount of photogenerated holes, which is distinctly inadequate for the quantity of organic material. In contrast, the obtained Q_net_ values are stable when the light intensities are stronger than 170 μW·cm^−2^. To enhance the stability of the quantity of light-excited photoelectron/hole pairs, a light intensity condition of 214 μW·cm^−2^ was selected for the subsequent experiments.

### 3.4. Selection of the Applied Bias

**Figure 6.**
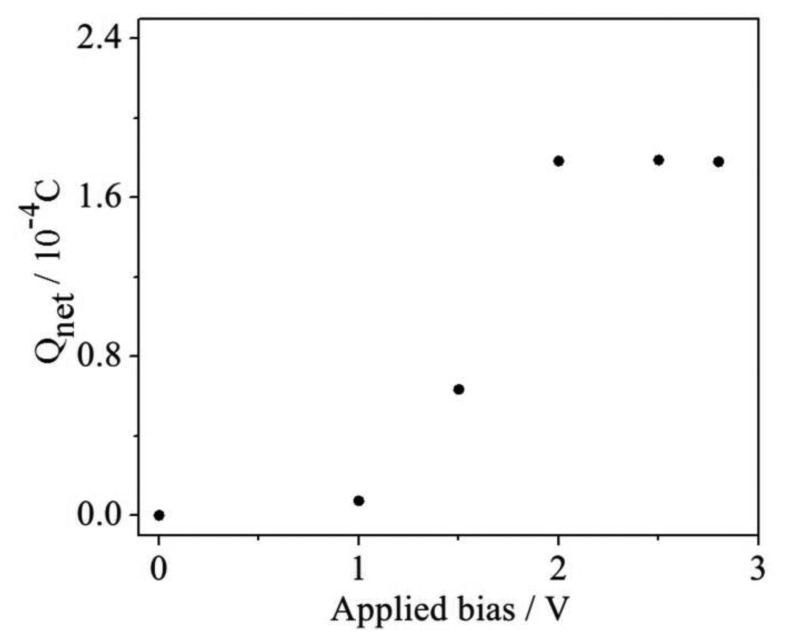
Effect of applied bias on Q_net_ determination.

In the phtotelectrocatalysis oxidation process, the photogenerated hole is a kind of very powerful oxidizing reagent for the degradation of organics. However, the recombination of the photogenerated electrons and holes is always a limiting factor for the degradation efficiency [13]. It has been reported that an applied bias could assist in transferring the photogenerated electrons to the external circuit, thus remarkably improving the organic compound degradation efficiency [9]. Therefore, a proper applied bias is necessary because the low voltage only supplies a weak transmission force so that the quick recombination of photogenerated electron/hole pairs occurs in a very short time. The effect of the applied bias on Q_net_ determination was investigated at different applied bias conditions and the results are shown in Figure 6, from which it can been seen that the determined Q_net_ values are much smaller when the applied bias values are lower than 2 V. When the applied bias is in the range of 2.0 to 2.8 V, the obtained Q_net_ values almost do not fluctuate. Thus, the condition of 2.0 V was selected for the next experiments.

### 3.5. Propose Method Stability and Accuracy

**Figure 7.**
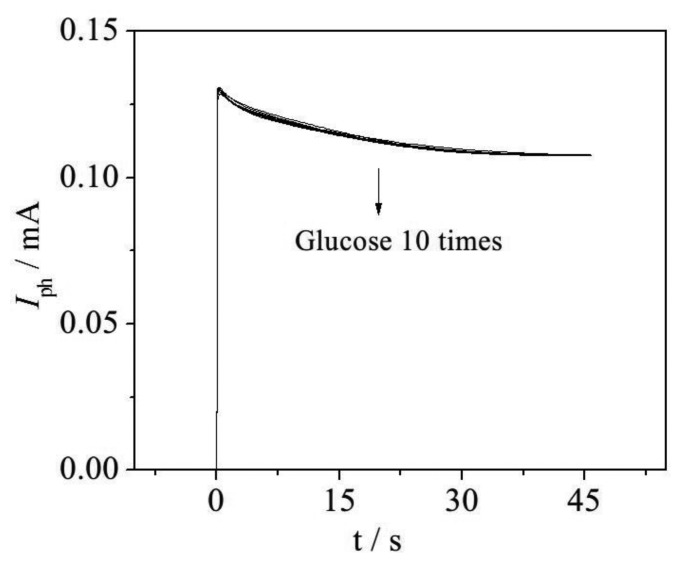
Continuous *I*–*t* curves obtained by WO_3_/W sensor.

To increase the stability and accuracy of the proposed method, samples containing 42 mg·L^−1^ ThCOD glucose were investigated. Response photocurrents from ten continuous experiments are shown in Figure 7, from which it can be seen that all of these *I*–*t* curves show a similar degradation process, as the curves almost coincide. In addition, the calculated relative standard deviation (RSD) is 3.60% for these ten samples, with a mean value of 42.23 mg·L^−1^. Furthermore, COD values determined by the photoelectrocatalysis method (PeCOD), e.g., with the WO_3_/W nanopores sensor, are shown in Table 1. The determined PeCOD values of glucose samples agreed well with the ThCOD, and the relative deviation (RD) of each determined value is smaller than 5%. The results illustrate that the proposed method is accurate and well reproducible.

### 3.6. Effect of pH

**Figure 8.**
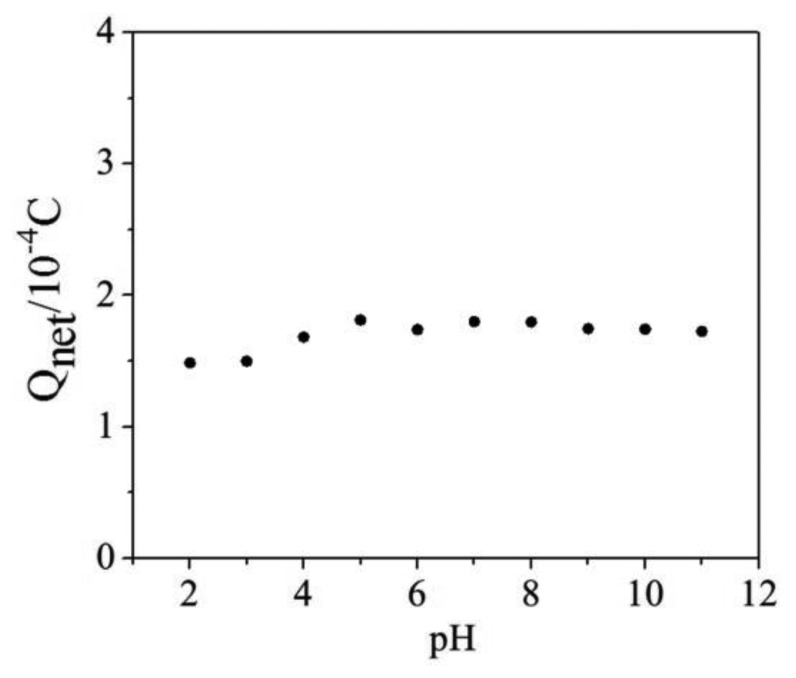
Effect of pH on Q_net_ determination.

The pH value of solution is a very important parameter in the phtotelectrocatalysis determination process, and the effect of pH on Q_net_ determination was investigated in this work. The Q_net_ values determined in a wide pH range of 2 to 11 are shown in Figure 8, where it can be seen that the Q_net_ values are stable in case of pH values ranging from 4 to 11 and the calculated RSD is 2.53% for eight samples with a mean value of 1.75 × 10^−4^ C. According to the national standards the pH values of surface water should between 6 and 9, which means the proposed method is applicable to detect these kinds of samples.

### 3.7. COD Determination

**Figure 9.**
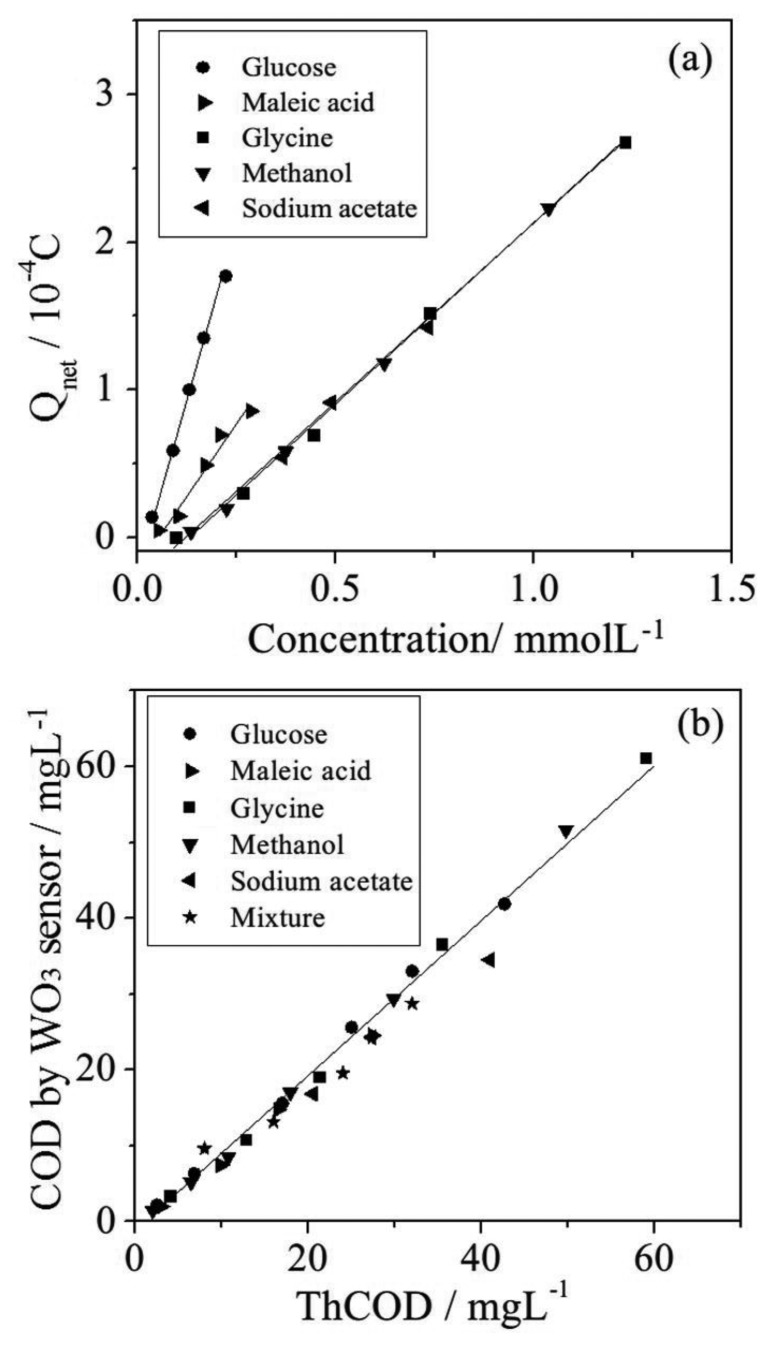
COD determination using a WO_3_/W nanopores sensor: (**a**) quantitative relationship between Q_net_ and the concentration of organic compounds; (**b**) The relationship between COD determined by the WO_3_ sensor and the theoretical COD value.

The COD values of various kinds of organics have been determined and the relationship between the Q_net_ values and the molar concentrations of individual organic compounds are shown in Figure 9a, which indicates the Q_net_ values are proportional to the concentration for each kind of organic compound investigated in this work. Figure 9b has been derived from Figure 9a by plotting the net charge against the theory values of COD. The plots reveal that all kinds of the compounds investigated in this work can be fitted by a straight line y = 1.013x − 1.654, (where *y* represents PeCOD obtained from phototelectrocatalysis oxidation by the WO_3_ sensor, *x* represents the ThCOD of the organics) with *R*^2^ = 0.98. For the different kinds of organics, the same Q_net_ has been obtained as long as the COD values of the organics are the same. It suggests that this method could be applicable to slightly polluted water such as groundwaters and the effluent of sewage treatment plants.

## 4. Conclusions

A sensor of a WO_3_/W nanoporous electrode combined with a thin layer reactor was proposed to determine COD, which is based on the relationship between COD and the quantity of charge captured applying the photoelelctrocatalysis method. The optimum conditions, including light intensity, applied bias and supporting electrolyte concentration, were investigated in photoelectrochemical experiments. The results showed a good relationship between PeCOD and the theoretical COD value, which suggests the application of this kind of sensor to slightly polluted water such as surface waters and sewage treatment plant effluents. In light of the advantages of the WO_3_ nanopores electrode, such as simplified operation, significantly shortened measurement times, reduce costs and absence of toxic and hazardous substances, the proposed WO_3_/W sensor could represent a new approach for COD determination.

## Figures and Tables

**Table 1.** Comparison of ThCOD and the PeCOD obtained by proposed method.

**No.**	**PeCOD (mg·L^−1^)**	**RD (%)**	**No.**	**PeCOD (mg·L^−1^)**	**RD (%)**
1	43.30	3.10	6	42.07	1.66
2	41.84	−0.37	7	40.31	−4.01
3	41.71	−0.69	8	40.97	−2.46
4	43.54	3.67	9	40.66	−3.20
5	44.04	4.86	10	43.88	4.49
